# Supplementary material for: Secreted Protein Acidic and Rich in Cysteine (SPARC) Polymorphisms in Response to Neoadjuvant Chemotherapy in HER2-Negative Breast Cancer Patients
Source: Biomedicines. 2023 Dec 6;11(12):3231. doi: 10.3390/biomedicines11123231 (PMC10741005; doi:10.3390/biomedicines11123231)
Supplement: Supplementary file 1 [file biomedicines-11-03231-s001.zip › biomedicines-2705676-supplementary.pdf]

Table S1. Associations between *SPARC* polymorphisms and clinicopathological characteristics

SNPs	Histologic grade		Hormone receptors		Ki-67 index		Tumor size		Lymph node status		Lymphovascular invasion		Perineural invasion	
	**OR (95%CI)**	***P*- value**	**OR (95%CI)**	***P*- value**	**OR (95%CI)**	***P*- value**	**OR (95%CI)**	***P*- value**	**OR (95%CI)**	***P*- value**	**OR (95%CI)**	***P*- value**	**OR (95%CI)**	***P*- value**
rs10065756														
C/C	Ref.		Ref.		Ref.		Ref.		Ref.		Ref.		Ref.	
A/C	1.05 [0.47;2.36]	0.912	0.73 [0.32;1.68]	0.471	0.81 [0.37;1.78]	0.609	0.56 [0.13;2.45]	0.465	1.17 [0.56;2.47]	0.677	0.53 [0.20;1.37]	0.195	0.48 [0.14;1.70]	0.268
A/A	2.06 [0.62;6.87]	0.258	0.69 [0.17;2.78]	0.637	0.53 [0.16;1.75]	0.314	0.68 [0.07;7.12]	0.736	1.67 [0.47;5.93]	0.453	0.90 [0.22;3.67]	0.913	0.00 [0.00;.]	0.170
rs12153644														
T/T	Ref.		Ref.		Ref.		Ref.		Ref.		Ref.		Ref.	
T/A	0.70 [0.31;1.62]	0.416	0.41 [0.18;0.97]	0.046	0.59 [0.25;1.40]	0.242	0.64 [0.12;3.47]	0.650	1.25 [0.57;2.70]	0.583	0.48 [0.18;1.24]	0.139	1.24 [0.35;4.39]	0.766
A/A	1.04 [0.32;3.33]	0.945	0.34 [0.08;1.35]	0.124	0.38 [0.12;1.22]	0.117	0.39 [0.05;3.01]	0.411	1.87 [0.57;6.19]	0.321	0.83 [0.23;3.07]	0.807	0.00 [0.00;.]	0.236
rs17718347														
T/T	Ref.		Ref.		Ref.		Ref.		Ref.		Ref.		Ref.	
T/C	1.06 [0.46;2.45]	0.890	0.54 [0.23;1.31]	0.182	0.89 [0.40;1.97]	0.773	0.64 [0.14;2.80]	0.575	1.16 [0.54;2.47]	0.712	0.70 [0.27;1.86]	0.490	0.74 [0.21;2.58]	0.653
C/C	2.08 [0.69;6.21]	0.207	1.10 [0.35;3.39]	0.866	0.72 [0.24;2.17]	0.570	1.06 [0.10;10.8]	0.986	1.93 [0.61;6.12]	0.280	1.40 [0.41;4.71]	0.596	0.44 [0.05;3.95]	0.519
rs19789707														
A/A	Ref.		Ref.		Ref.		Ref.		Ref.		Ref.		Ref.	
A/G	0.48 [0.21;1.12]	0.095	0.66 [0.26;1.64]	0.383	1.16 [0.52;2.60]	0.725	0.67 [0.16;2.82]	0.611	0.85 [0.38;1.90]	0.696	0.47 [0.19;1.21]	0.124	1.10 [0.29;4.14]	0.906
G/G	0.64 [0.22;1.85]	0.427	1.55 [0.54;4.42]	0.429	1.75 [0.58;5.26]	0.336	. [;.]	0.259	0.64 [0.23;1.74]	0.391	0.21 [0.04;1.04]	0.043	0.89 [0.15;5.25]	0.931
rs19789707[a]														
A/A o A/G	Ref.		Ref.		Ref.		Ref.		Ref.		Ref.		Ref.	
G/G	0.95 [0.36;2.51]	0.941	1.95 [0.76;4.95]	0.177	1.61 [0.59;4.37]	0.367	. [.;.]	0.141	0.70 [0.29;1.70]	0.439	0.32 [0.07;1.45]	0.121	0.84 [0.17;4.11]	0.888
rs2347128														
C/C	Ref.		Ref.		Ref.		Ref.		Ref.		Ref.		Ref.	
C/G	1.35 [0.52;3.55]	0.555	1.24 [0.47;3.29]	0.674	0.79 [0.32;1.95]	0.619	0.41 [0.04;3.79]	0.476	0.48 [0.20;1.18]	0.112	1.96 [0.58;6.61]	0.290	0.55 [0.15;2.06]	0.398
G/G	2.07 [0.73;5.87]	0.182	1.21 [0.41;3.61]	0.740	0.74 [0.27;2.02]	0.566	0.22 [0.02;2.09]	0.195	0.74 [0.27;2.02]	0.566	2.77 [0.76;10.0]	0.127	0.38 [0.07;2.08]	0.284
rs3210714														
G/G	Ref.		Ref.		Ref.		Ref.		Ref.		Ref.		Ref.	
G/A	0.89 [0.35;2.22]	0.801	0.64 [0.26;1.63]	0.364	0.83 [0.35;1.97]	0.680	0.33 [0.04;3.08]	0.362	0.55 [0.24;1.28]	0.174	3.74 [0.99;14.1]	0.043	0.69 [0.19;2.57]	0.596

A/A	1.69 [0.64;4.49]	0.307	0.87 [0.31;2.40]	0.798	0.87 [0.32;2.32]	0.778	0.19 [0.02;1.76]	0.142	1.11 [0.41;2.99]	0.838	4.56 [1.12;18.5]	0.031	0.45 [0.08;2.48]	0.389
rs4958487														
A/A	Ref.		Ref.		Ref.		Ref.		Ref.		Ref.		Ref.	
A/G	0.67 [0.28;1.56]	0.361	0.51 [0.22;1.20]	0.133	0.84 [0.36;1.97]	0.695	0.29 [0.03;2.50]	0.266	1.98 [0.89;4.38]	0.097	0.52 [0.20;1.37]	0.199	1.50 [0.37;6.01]	0.599
G/G	1.00 [0.32;3.08]	0.993	0.30 [0.07;1.19]	0.083	0.64 [0.21;1.96]	0.449	0.25 [0.02;2.93]	0.317	4.47 [1.27;15.7]	0.017	0.68 [0.19;2.52]	0.592	0.60 [0.06;6.16]	0.731

SNP: single-nucleotide polymorphism; OR: odds ratio; CI: confidence interval; Ref.: reference

Histologic grade (G3 vs G1/G2); hormone receptors (estrogen receptors and/or progesterone receptors; positive vs negative); ki-67 index (≥14% vs <14%); tumor size (>2cm vs ≤2cm); lymph node status (N+ vs N0); lymphovascular invasion (positive vs negative); perineural invasion (positive vs negative)

[a]recessive model
